# Predictors of microvascular complications in patients with type 2 diabetes mellitus at regional referral hospitals in the central zone, Tanzania: a cross-sectional study

**DOI:** 10.1038/s41598-024-55556-x

**Published:** 2024-02-29

**Authors:** Wilfred B. Shillah, James J. Yahaya, Emmanuel D. Morgan, Deogratius Bintabara

**Affiliations:** 1 Department of Community Medicine, School of Medicine and Dentistry, University of Dodoma, Dodoma, Tanzania; 2 Department of Pathology, School of Health Sciences, Soroti University, P. O. Box 211, Soroti, Uganda

**Keywords:** Type 2 diabetes mellitus, Microvascular complications, Predictors, Endocrinology, Medical research

## Abstract

Microvascular complications encompass a group of diseases which result from long-standing chronic effect of diabetes mellitus (DM). We aimed to determine the prevalence of microvascular complications and associated risk factors among patients with type 2 diabetes mellitus (T2DM). A cross-sectional analytical hospital-based study was conducted at Singida and Dodoma regional referral hospitals in Tanzania from December 2021 to September 2022. A total of 422 patients with T2DM were included in the analysis by determining the prevalence of microvascular complications and their predictors using multivariable logistic regression analysis. A two-tailed *p* value less than 0.05 was considered statistically significant. The prevalence of microvascular complications was 57.6% (n = 243) and diabetic retinopathy was the most common microvascular complication which accounted for 21.1% (n = 89). Having irregular physical activity (AOR = 7.27, 95% CI = 2.98–17.71, *p* < 0.001), never having physical activity (AOR = 2.38, 95% CI = 1.4–4.01, *p* = 0.013), being hypertensive (AOR = 5.0, 95% CI = 2.14–11.68, *p* = 0.030), having T2DM for more than 5 years (AOR = 2.74, 95% CI = 1.42–5.26, *p* = 0.025), being obese (AOR = 2.63, 95% CI = 1.22–5.68, *p* = 0.010), and taking anti-diabetic drugs irregularly (AOR = 1.94, 95% CI = 0.15–0.77, *p* < 0.001) were the predictors of microvascular complications. This study has revealed a significant proportion of microvascular complications in a cohort of patients with T2DM. Lack of regular physical activity, being obese, taking anti-diabetic drugs irregularly, presence of hypertension, and long-standing duration of the disease, were significantly associated with microvascular complications.

Diabetes mellitus (DM) is a heterogeneous metabolic disorder characterized by common feature of chronic hyperglycemia with disturbance of carbohydrate, fat and protein metabolism^1^. The disease is clinically categorized into two types; type 1 diabetes mellitus (T1DM) and type 2 diabetes mellitus (T2DM) depending on the age of the individual at onset. T2DM which affects adults, represents approximately 98% of all cases of DM diagnosed globally, nevertheless, this proportion differs considerably among countries. Based on the International Diabetes Federation (IDF) report of the year 2021, the world incidence of T2DM among adults was 536.6 million people (10.5%), and also the report projected that, there would be 783.2 million people (12.2%) living with diabetes worldwide by 2045^2^. The incidence of T2DM of 4.7% which was reported in the sub-Saharan Africa (SSA) region is quite low, although this incidence varies by country, with the highest number of people with T2DM are living in more affluent countries^3^. Tanzania is one of the countries in the SSA region with the highest prevalence of T2DM and recently it was reported that the prevalence of T2DM was ranging between 9 and 11%^4^. T2DM being a chronic disease, can cause both macrovascular as well as microvascular complications including end-stage renal disease, adult-onset blindness, and non-traumatic lower extremity amputation^5^.

Microvascular complications are the major outcome of T2DM progression, which affect significantly the quality of life of patients, contribute to substantive economic burdens to the health care system and increase mortality rate as well as morbidity^6^. Microvascular complications of T2DM do not occur before 5–20 years after onset of the disease^7^. The pathogenic mechanisms which accelerate the development of the microvascular complications is not exactly known, however, possible attributable factors include but not limited to hyperlipidemia, reduced high density lipoprotein (HDL) level, non-enzymatic glycosylation, increased platelet adhesiveness, and hypertension in patients with DM^8–10^. Approximately 35% of patients with T2DM in Africa develop microvascular complications within 3 years of diagnosis and 18% of them die of such complications after 20 years of diagnosis^11,12^. Damage to small blood vessels in various body parts as a result of prolonged duration of the disease and poor control of hyperglycemia^13^. There are various types of microvascular complications related to T2DM such as neuropathy, retinopathy, and nephropathy^14^. Zhang et al.^15^ reported a prevalence of up to 40% of patient with T2DM in China who end up developing microvascular complications. Also, Seid et al.^16^ reported 37.9% of patients with T2DM had at least one type of microvascular complications in a study which was conducted in Ethiopia. In another study which was done in the United States of America, it was found that the prevalence of microvascular complications among patients with T2DM was 19.0%^17^. In Tanzania, the prevalence of microvascular complications among patients with T2DM was found to be 50.2%^18^.

The attributable risk factors to microvascular complications vary considerably. For example, in Africa they include financial health expenditure, poor medical facilities and lack of adequate diabetes services in both urban and rural areas^19^. In the study of Msanga et al.^20^ which was done it Tanzania it was reported that poor glycaemic control, old age, and longer duration of DM. Yau et al.^21^ also reported that, high blood pressure is associated with increased risk of developing microvascular complications among patients with T2DM. Identifying the possible risk factors for microvascular complications among patients with T2DM would significantly in way or another help to prevent the development and progression of such complications. Therefore, we aimed to investigate the prevalence and associated risk factors of microvascular complications in a cohort of patients with T2DM.

## Methods

### Study design, setting, and duration

This was a cross-sectional analytical hospital-based study which involved quantitative approach for collection of the required data. The study was conducted at two different regional referral hospitals (Dodoma and Singida) which are found in the central zone of Tanzania. The study was conducted for 10 months from December 2021 to September 2022. The two health facilities are located in two different fastest growing places in the country with more increased urbanization which contributes to sedentary life and other related risk factors for DM including alcoholism and smoking.

### Study population and recruitment process

We included both inpatient and outpatient adults with T2DM who were attending diabetes clinic at the selected health facilities. The criteria for inclusion in the study were as follows: being adult with a confirmed diagnosis of T2DM, being diabetic for not less than 3 years, and willing to sign informed consent. Those who refused to sign informed consent, pregnant women, and being seriously ill all were excluded from the analysis.

### Sample size estimation and sampling method

The sample size was calculated by considering a single population proportion formula: n = z^2^ p (100 − p)/e^2^^22^ assuming a standard normal variables (z score) of 1.96 at 95% confidence interval (CI), margin error (e) of 5%, and a proportion (p) of microvascular complications of 50.8% among patients with T2DM from a previous study^23^ Accordingly, the sample size became 384. Then we considered attrition rate of 10%, making the total sample size to be 422. The patients were recruited using convenience sampling method after meeting the inclusion criteria. We selected 184 and 238 patients from Singida and Dodoma regional referral hospitals, respectively.

### Measurement of variables

#### Physical activity

Physical activity was considered as categorical variable in which it was grouped into three categories; regularly, irregularly, and never. Regular and irregular physical activity referred to abiding to performing physical activity every day for given length of time and vice versa, respectively^24^. The group of physical activity was self-reported and patients were asked to report regarding their habits of doing physical activity since when they were diagnosed with T2DM.

#### Dietary intake

Proper diet was defined as the diet with more non-starchy vegetables, fewer added sugars and refined grains, less than 2 gm of fiber per serving and contains whole foods instead of highly processed foods as much as possible^25^. Therefore, patients who reported to be taking any diet with missing or decreased or increased amount of any of the stated ingredients was considered to be improper.

#### Glycemic control

Good glycemic control was defined as an average of three consecutive fasting blood glucose measurement between 80 and130 mg/dL. Poor glycemic control was defined as patients who had average blood glucose measurements on three consecutive visits > 130 or < 70 mg/dL^26^. All subjects were categorized into three groups; normal, hypoglycemic, and hyperglycemic. Then both subjects with hypoglycemic and hyperglycemic statuses were termed as having poor glycemic control.

#### Body mass index

Anthropomorphic variables including height and weight of the patients were used to determine the body mass index (BMI) of each individual by taking weight of the person in kg and dividing with square root of height in metres; kg/m^2^ as it was done in a previous study^18^. Categorization of BMI was in accordance to World Health Organization (WHO)^27^ as normal weight (20 to 24.9 kg/m^2^), overweight (≥ 25 kg/m^2^), and obesity (> 30 kg/m^2^).

#### Blood pressure

Both systolic blood pressure and diastolic blood pressure were measured from the left arm at the level of the heart using a mercury-based sphygmomanometer after the patients had taken a rest up to 30 min as previous^28^. For those study subjects with SBP ≥ 140 mm of mercury (mmHg) and DBP ≥ 90 mmHg, blood pressure was measured again and finally the average value was taken.

#### Diabetic neuropathy

We detected diabetic neuropathy if either touch sensation detection using 10 gm monofilament test indicated a score of < 7 out of 10 or vibration sensation detection using biothesiometer was abnormal.

#### Diabetic retinopathy

Diabetic retinopathy was diagnosed using detailed fundus examination by retinoscope.

#### Diabetic nephropathy

Diabetic nephropathy was diagnosed based on the urine albumin to creatinine ratio (uACR) of a single urine spot sample. A ratio of > 2.5 mg/mol and > 3.5 mg/mol was diagnostic of diabetic nephropathy in males and females, respectively. All the assessed microvascular complications as it was done in the previous study by Arambewela et al.^23^.

### Data collection procedures

Data were collected using a face to face interviewer administered semi-structured questionnaire. We adapted the questionnaire from a previous study which was done in Sri Lanka^23^. The questionnaire was exported into Kobo Toolbox for simplifying the process of data collection and helping to make the collected data to be available online. Before data collection, the questionnaire was validated by pre-testing it among 30 patients with T2DM from a health facility different from the study sites. The data were collected in a separate room for maintaining privacy during diabetes clinics. All researchers and two research assistants (medical registrars) were responsible for the data collection. Cronbach’s alpha of 0.83 for internal consistency of the validated questionnaire was acceptable.

### Data analysis

Data were analyzed using SPSS version 25.0. All categorical and continuous variables were summarized as frequency and percentages and mean ± standard deviation (SD), respectively. Binary logistic regression analysis was used to assess the predictors of chronic complications using enter method. All variables with *p* ≤ 0.2 in bivariate logistic regression analysis were fitted into multivariable logistic regression analysis. A two-tailed *p* < 0.05 was considered statistically significant.

### Ethical approval

We obtained ethical approval from the Institutional research committee of the University of Dodoma (reference MA.84/261/04/A). Additionally, permission was obtained from the medical officers in-charge of the two regional referral hospitals (Dodoma: reference DA/122/467/01 G/04) and (Singida: reference BA.381/391/01 F/232). We also confirm that all methods were performed in accordance with the relevant guidelines and regulations.

## Results

### Baseline characteristics of the study participants

The Baseline characteristics of the study patients are presented in Table 1. The mean age of the study participants was 54.4 ± 12.2 years and most of the participants 52.8% (n = 228) were of age between 46 and 60 years. Of the 422 patients analyzed in the present study, most of the participants 58.8% (n = 248) were males. Over one-third 38.2% (n = 161) of the participants had attained secondary school. Regarding body mass index (BMI), 20.1% (n = 85) of the patients were obese (≥ 30 kg/m^2^).

**Table 1:** Baseline characteristics of the study subjects (N = 422).

Variables	Frequency (n)	Percentage (%)
Age (years)		
18–39	40	9.5
40–59	223	52.8
≥ 60	159	37.7
Sex		
Male	248	58.8
Female	174	41.2
Residence		
Urban	279	66.1
Rural	143	33.9
Religion		
Christian	241	57.1
Muslims	160	37.9
Traditional	21	5.0
Marital status		
Single	41	9.8
Married/cohabiting	204	48.3
Divorced/separated	128	30.8
Widow/widower	49	11.6
Education level		
No formal education	64	15.2
Primary	133	31.5
Secondary	161	38.2
Tertiary	64	15.2
Occupation		
Employed	111	26.3
Self-employed	193	45.7
Unemployed	118	28.0
Family income (TZS)		
< 150, 000	29	6.9
150,000–499,999	137	32.5
500,000–1,000,000	159	37.7
> 1,000,000	97	23.0
BMI (kg/m^2^)		
Normal (18–24.9)	254	60.2
Underweight (< 18)	1	0.2
Overweight (25–29)	82	19.4
Obese (≥ 30)	85	20.1

### Clinical characteristics and lifestyle behaviors of the study subjects

The vast majority 68% (n = 287) of the patients had poor glycemic control. The duration of T2DM was more than 5 years in over one-third 35.8% (n = 151) of the patients. It was also found that majority 73.7% (n = 311) of the patients were on single oral anti-diabetic drugs (metformin). Also, almost one-third 29.9% (n = 126) of the participants were hypertensive (Table 2).

**Table 2:** Clinical characteristics and lifestyle behaviors of the study subjects (N = 422).

Variables	Frequency (n)	Percentage (%)
Blood pressure (mmHg)		
Normal (90–120/60–80)	205	48.6
Pre-hypertension (120–129/81–85)	91	21.6
Hypertension stage 1 (130–139/86–89)	64	15.2
Hypertension stage 2 (> 140/90)	62	14.7
Duration of illness (years)		
≤ 5	271	64.2
> 5	151	35.8
Comorbidities		
Yes	171	40.5
No	251	59.5
Type of comorbidities		
Hypertension	126	29.9
HIV/AIDS	8	1.9
CKD	11	2.6
PUD	13	3.1
Heart failure	10	2.4
Epilepsy	3	0.7
Treatment		
Single oral agent (metformin)	311	73.7
Combined oral agents (metformin & glibenclamide)	99	23.5
Injectable (insulin)	3	0.7
Injectable and oral agents (metformin, insulin)	9	2.1
Habit of taking anti-diabetic drugs		
Regularly	358	84.8
Irregularly	64	15.2
Glycemic control		
Good	135	32.0
Poor	287	68.0
Physical activity		
Regularly	52	12.3
Irregularly	161	38.2
Never	208	49.5
Dietary intake		
Proper	93	22
Improper	329	78
Smoking habit		
Yes	42	10.0
No	380	90.0
Alcohol intake		
Yes	82	19.4
No	340	80.6

### Prevalence of microvascular complications

Of all the participants, 57.6% (n = 243) of the study participants had at least one microvascular complication. Diabetic retinopathy was the most common microvascular complication which accounted for 21.1% (n = 89) followed by peripheral neuropathy which was found in 11.1% (n = 47) of all the patients. Two participants (0.5%) had three microvascular complications (Table 3).

**Table 3:** Prevalence of microvascular complications (N = 422).

Chronic complications	Frequency (n)	Percentage (%)
Diabetic neuropathy	47	11.1
Diabetic nephropathy	37	6.4
Diabetic retinopathy	89	21.1
Diabetic neuropathy with nephropathy	26	6.2
Diabetic neuropathy with retinopathy	14	3.3
Diabetic nephropathy with retinopathy	5	1.2
Diabetic foot ulcer	23	5.5
Diabetic neuropathy, nephropathy and retinopathy	2	0.5
Without microvascular complications	169	42.4

### Predictors of microvascular complications among patients with T2DM

Only variables with *p* ≤ 0.2 after performing univariate analysis were further fitted into multivariable logistic regression analysis which included age, sex, education level, family income, BMI, BP, physical activity, and duration of illness. Physical activity, BP, BMI, duration of illness, and habit of taking anti-diabetics remained the independent predictors of microvascular complications. Patients who reported to be doing physical activity irregularly (95% CI 2.98–17.71, *p* < 0.001) and those who never engaged in physical activity (95% CI 1.41–4.01, *p* = 0.013) were 7 times and 2 times more likely to develop microvascular complications, respectively. Also, patients who were hypertensive they were 5 times more likely to develop microvascular complications than patients who were not hypertensive (AOR = 5.0, 95% CI 2.14–11.68, *p* = 0.030).

Patients who were obese, they were almost 3 times more likely to have microvascular complications than non-obese patients (95% CI 1.22–5.68, *p* < 0.010). Increased duration of T2DM for more than 5 years was significantly associated with almost a threefold increased chance of developing microvascular complications compared to having the disease for not more than 5 years (95% CI 1.42–5.26, *p* = 0.025). Additionally, patients who had a habit of taking anti-diabetics irregularly were almost 2 times likely to develop microvascular complications compared to patients who had a habit of taking their anti-diabetics regularly. (95% CI 0.15–0.77, *p* < 0.001). Other variables were not statistically significant (Table 4).

**Table 4:** Multivariate logistic regression analysis for predictors of microvascular complications among patients with T2DM.

Variables	Univariate analysis	Multivariate analysis
	COR (95% CI), *p*-value	AOR (95% CI), *p*-value
Age (years)		
18–39	1	1
40–59	1.63 (0.30–5.34), 0.070	0.70 (0.27–1.84), 0.064
≥ 60	1.58 (0.14–2.41), 0.083	0.74 (0.40–1.35), 0.550
Sex		
Male	1	1
Female	0.75 (0.51–1.12), 0.162	1.15 (0.71–1.88), 0.309
Education level		
Informal	1	1
Primary	0.39 (0.83–1.92), 0.173	0.60 (0.76–3.39), 0.078
Secondary	0.35 (0.99–3.37), 0.054	0.67 (0.80–3.49), 0.091
Tertiary	0.24 (0.89–3.75), 0.103	0.94 (0.80–4.68), 0.082
Family income (TZS)		
< 150,000	1	1
150,000–499,999	0.83 (0.62–5.98), 0.075	0.46 (0.62–7.98), 0.601
500,000–999,999	0.41 (0.22–2.33), 0.309	0.33 (0.70–2.84), 0.084
≥ 1,000,000	0.79 (0.45–11.78), 0.103	0.21 (0.53–0.79), 0.407
BMI (kg/m^2^)		
Non-obese (< 30)	1	1
Obese (≥ 30)	5.27 (0.12–0.45), < 0.001	2.63 (1.22–5.68), 0.010
Blood pressure (mmHg)		
Non-hypertensive (< 140/ < 90)	1	1
Hypertensive (≥ 140/ ≥ 90)	8.72 (4.23–17.96), 0.040	5.0 (2.14–11.68), 0.030
Duration of illness (years)		
≤ 5	1	1
> 5	1.98 (0.12–0.32), < 0.001	2.74 (1.42–5.26), 0.025
Physical activity		
Regular	1	1
Irregular	4.11 (0.32–0.69), 0.02	7.27 (2.98–17.71), < 0.001
Never	1.66 (0.56–3.78), 0.041	2.38 (1.41–4.01), 0.013
Habit of taking anti-diabetics		
Regularly	1	1
Irregularly	2.33 (0.58–1.44), 0.012	1.94 (0.15–0.77), < 0.001

## Discussion

Microvascular complications are significantly associated with visual, renal and neurological pathologies in patients with T2DM, and all together result in high morbidity, mortality, and negative socio-economic consequences^16^. Identification of factors associated with microvascular complications is of paramount importance in prevention of their irreversible consequences and improvement of health of the patients.

In the present study, the percentage of patients with at least one microvascular complication was in line with the findings in the studies done in Bangladesh (50.4%)^29^, China (57.5%)^30^, India (52.1%)^31^, and Tanzania (50.2%)^18^. However, lower prevalence of microvascular complications among patients with T2DM of 41.5%, 38.5%, 35.3%, 35.4%, 41.6%, and 41.9% which was reported in the Jimma, Ethiopia^32^, Metu, Ethiopia^33^, Ghana^34^, Saudi Arabia^35^, Brazil^36^, and Tanzania^20^, respectively. On the other hand, the prevalence of microvascular complications observed in this study was lower than 61%, 61.6%, 69%, 68%, and 77% which were reported in Gurage, Ethiopia^37^, Kuwait^38^, India^39^, Greenland^40^ and USA^41^. The difference in prevalence of microvascular complications observed might be related to variation in accessibility and status of health facilities in the respective settings, patient’s adherence to anti-diabetic drugs and practices of the patients. Studies have shown that high medication adherence among patients with T2DM helps to have good glycemic control, which in turn prevents development of complications including microvascular complications^42,43^.

Diabetic retinopathy was the most prevalent type of microvascular complication in this study. This is similar to other studies^35,41,44,45^, in which diabetic retinopathy was also the most prevalent microvascular complication, however, in other studies^13,30,31,33,40,46^ diabetic neuropathy was reported to be the most common microvascular complication among patients with T2DM. Another study which was done in Tanzania among patients with T2DM showed that, almost half of the study participants had diabetic retinopathy^18^. However, a low prevalence of diabetic retinopathy was found among children and adolescents in a study which was done in Tanzania^20^. The variation of a number of clinical characteristics in patients included in the studies such as glycemic control and disease duration, could help to explain the discrepancy of the prevalence of the microvascular complications in various studies. This is because patients with DM for a long duration of the disease, poor glycemic control, and hypertension have increased risk of developing diabetic retinopathy^47^.

Long duration of diabetes among patients for more than 5 years was a predictor of microvascular complications in this study similar to the observation in the studies done elsewhere^7,35,48,49^. The longer the duration of diabetes since diagnosis has been linked to increasing odds of development of not only microvascular complications but also macrovascular complications such as cardiovascular diseases, cerebrovascular diseases and diabetic foot ulcer^50,51^. It is imperative for patients with diabetes for more than 5 years to be always to be under close monitoring for possible complications, this helps to ensure that such complications are detected as early as possible for optimal outcomes of the patients.

Hypertension was another predictor of microvascular complications in this study, which is similar to the findings in previous studies^13,30,35,49^. Other studies published previously have also reported a significant association of hypertension with development of microvascular complications among patients with T2DM^13,30,35,49^. Hypertension has been found to be the most common comorbidity among patients with T2DM is hypertension and it is likely to increase the chance of developing microvascular complications and even worsen their clinical course^52^. However, some studies have reported contradicting findings regarding the association of the two variables, for example, Bonora et al. reported no association between hypertension and microvascular complications in a cohort of patients with T2DM^7^.

Irregular intake of anti-diabetic drugs as well as low medication adherence among patients with DM, are a standalone and significant contributing factor for development of both macrovascular and microvascular complications in DM. In this study, patients who reported to have been taking their ant-diabetic drugs irregularly, had increased odds of developing microvascular complications compared to other patients who were having a habit of taking their medications regularly. Also, similar observation was reported in other studies^50,53^. In one study it was found that, intensive treatment of patients with preserved insulin secretion had 35% and 23% reductions in risk of retinopathy and nephropathy^54^.

Regarding association of BMI with microvascular complications, it was found that, there was increased risk of developing microvascular complications in patients with obesity compared with patients who were not obese. Other studies have also reported similar findings^7,45,55^. It has been reported that there is a linear association between high BMI and increased odds developing microvascular diseases, such as retinopathy, nephropathy, and neuropathy, in patients with T2DM^56,57^. Furthermore, it was observed that, physical inactivity was also significantly associated with increased odds of being diagnosed with microvascular complications. Physical activity helps patients with T2DM to be active, this prevents development of both macrovascular and microvascular complications through inhibition of other risks including obesity and hypertension^58,59^.

### Study limitations

This study encountered some limitations. Considering the nature of the study that it was hospital-based; therefore, our results cannot be generalized. Fasting blood sugar test was used to measure the blood sugar of the patients instead of glycated haemoglobin (HbA1C) which usually gives a wide span of blood sugar of the individual due to financial constraints.

## Conclusion

The results our study have shown that a significant number of patients with T2DM in Tanzania end-up developing microvascular complications. Also, this study has shown that factors such as obesity, hypertension, being physically inactive, and irregular intake of anti-diabetic drugs in patients with diabetes, all carry high risk of developing microvascular complications such a retinopathy, peripheral neuropathy, and nephropathy. Therefore, modification of such lifestyle practices would help to reduce the occurrence of such complications in the population of patients with T2DM significantly.

## Data Availability

The dataset used for this study is restricted by the Research Ethical Committee of the institution detail due to containing sensitive patient information, however, it can be accessed upon reasonable request from the Directorate of Research Publication, and Consultancy (DRPC), University of Dodoma, P. O. Box 259, Dodoma, Tanzania. drpc@udom.ac.tz.

## Abbreviations

DM: Diabetes mellitus
T2DM: Type 2 diabetes mellitus
HbA1C: Glyated haemoglobin
BMI: Body mass index
BP: Blood pressure
